# Determinants of home care utilization among the Swedish old: nationwide register-based study

**DOI:** 10.1007/s10433-021-00669-9

**Published:** 2021-12-17

**Authors:** Anders Brändström, Anna C. Meyer, Karin Modig, Glenn Sandström

**Affiliations:** 1 Historical Demography, Department of Historical, Philosophical and Religious Studies, Umeå University, Umeå, Sweden; 2 Institute of Environmental Medicine, Unit of Epidemiology, Karolinska Institute, Solna, Sweden; 3 Stockholm University Demography Unit (SUDA), Stockholm University and Historical Demography, Department of Historical, Philosophical and Religious Studies, Umeå University, Stockholm, Sweden

**Keywords:** Home care, Living arrangements, Health, Municipal care, Sweden

## Abstract

Since the 1990s, Sweden has implemented aging-in-place policies increasing the share of older adults dependent on home care instead of residing in care homes. At the same time previous research has highlighted that individuals receive home care at a higher age than before. Consequently, services are provided for a shorter time before death, increasing reliance on family and kin as caregivers. Previous studies addressing how homecare is distributed rely primarily on small surveys and are often limited to specific regions. This study aims to ascertain how home care services are distributed regarding individual-level factors such as health status, living arrangements, availability of family, education, and socioeconomic position. To provide estimates that can be generalized to Sweden as a whole, we use register data for the entire Swedish population aged 65 + in 2016. The study's main findings are that home care recipients and the amount of care received are among the oldest old with severe co morbidities. Receiving home care is slightly more common among women, but only in the highest age groups. Childlessness and socioeconomic factors play a small role in who receives home care or not. Instead, the primary home care recipients are those older adults living alone who lack direct support from family members residing in the same household.

**Supplementary Information:**

The online version contains supplementary material available at 10.1007/s10433-021-00669-9.

## Introduction

Home care services to older adults are provided by Sweden’s 290 municipalities and are regulated through the Social Services Act (Socialtjänstlag 2001:453). A central paragraph is Ch. 1. §1, with its equity and social justice concept. Older adults are entitled to care based on their needs regardless of where they live in the country or other individual characteristics such as age, sex, and socioeconomic position (see also Davey et al. 2006). With a high degree of freedom, it is then for each of the municipalities to organize and finance how these goals will be met (Hedman et al., 2007). However, increasing budget constraints at the municipal level (Plesner 2020) have pushed the chances of getting home care to higher age groups and those with more severe conditions (Parker et al. 2005; Szebehely 2005; Larsson 2006).

Aging in place policies has resulted in sharp declines in older adults who live in old-age care facilities.^1^Policies have instead focused on support and services through home care. At the peak of institutionalized care in Sweden in 1975, 9 percent of the population aged 65 + were in such facilities (Sundström et al., 2011).^2^ At present, about 4 percent of older adults live in institutions (Johansson et al. 2018), which reflects Sweden’s aging-in-place policy of replacing institutional care with home care (Rodrigues et al. 2012). This change, it is argued, is compensated by a re-familization of home care where family and other kin take on greater responsibilities (Johansson et al. 2018; Ulmanen and Szebehely 2015; Larsson 2006; Larsson et al. 2006; Sundström et al. 2006).

Re-familization is, however, not a straightforward concept. Several studies, mainly based on SHARE data,^3^ underline that the family care of older parents has always played and still plays a significant role in Scandinavia/Northern Europe (See, for instance, Fihel et al. 2021; Albertini and Kohli 2013; Hank and Buber 2009; Bordone 2009). Data from Sweden 2009–2010 show that 31 percent of the 70 + population relied on extra help from non-cohabiting family or friends, despite having home care. (Ulmanen and Szebehely, 2015).

According to Fihel et al., proximity to family and close kin plays a vital role in more extensive informal care, but only if parents live within a kilometer. Consequently, the number of hours a family can spend on care of their parents decreases with distance (2021). However, how home care is distributed to older adults and the role played by family caregivers is complicated to disentangle. For instance, informal care can take many shapes and forms depending on the specific situation and may differ from time to time.

When aging-in-place policies prioritize individuals in higher age groups and with substantial care needs, family support might be shifting from including ADL to almost exclusively IADL.^4^ If we are experiencing such a policy-induced shift affecting families' engagement, the distance to family caregivers outside the household might play a lesser role today than before. For instance, helping older parents negotiating with authorities, banking, and other practical issues could be done over the telephone or computer, regardless of proximity. Hypothetically, such help from a distance could increase the possibility of parents getting home care and the number of hours they receive rather than the opposite.

A general concern is that studies on home care allocation using representative national samples are lacking. Most of our knowledge builds on relatively small, local samples or surveys, often including less than 1000 individuals in the relevant age groups. Limited sample sizes have also made it difficult to assess whether are considerable differences between municipalities in care allocation. We need larger datasets given that individual factors such as age, health status, economy, and socioeconomic position must be considered as well (see discussion in Savla et al. 2008).

Regional variations in home care must be considered since Swedish municipalities face very different challenges. For instance, in 2016, dependency ratios spanned from 50 to almost 110, and municipal taxes, which finance home care, extended from 29 to 35 percent (Statistics Sweden 2021). There are also marked urban–rural differences in population density, making it more challenging to provide services in sparsely populated municipalities. This is especially true in the Northern parts of Sweden. Therefore, studies based on single or small clusters of municipalities will consequently struggle with representativeness. As far as we know, our study is the first to use linked register data for the entire non-institutionalized Swedish population in 2016 aged 65 and older (N 1,900,546), thereby taking regional differences and individual characteristics of the recipients into consideration.

Previous research has shown that the educational level of older adults contributes to better health, especially among the “younger old” (Sabater et al. 2020; Leopold 2018; Torssander 2013). The Swedish system should be built on the assessment of the need for care. However, there is a risk that other factors contribute to the municipal decision of providing both home care per se and the number of hours provided. Factors such as education and the recipients' income may influence the amount of care received—aspects that are not directly linked to needs based on health. The municipalities heavily subsidize home care, but it still comes with a monthly fee for the users—costs that might inhibit older adults with lower income from applying for these services.^5^ A high-income level can make it possible for recipients to directly pay for extra hours of services (Arpino et al. 2018; Batljan et al. 2009; Neuberger and Preisner 2018). A higher level of education could facilitate communication with authorities and increase the knowledge about legal rights to municipal care.

Our main objectives are to analyze how home care services distribute and how such services, measured in mean hours of care received, are allocated. We will use two indicators. First, we determine the probability of receiving home care dependent on the individual's demographic, socioeconomic, and health-related characteristics. Second, we analyze the mean amount of care in hours per month that an individual receives only among home care recipients, depending on personal characteristics. Since concerns are raised about whether municipalities can provide equitable home care because of significant differences in economy and demography (Plesner 2020), we will assess the variation in care allocation across municipalities when controlling for individual-level determinants.

To assess the potential impact of family caregivers, we estimate differences between individuals who live alone and those who cohabitate. We also estimate differences between childless individuals and those having children. The expectation is that access to family caregivers reduces the amount of care provided.

As gender roles between men and women may differ in fulfilling a caregiving role, we also test for interactions between living arrangements and sex.^6^ The hypothesis is that living with a spouse is potentially more critical for frail men than women, as women would be more inclined to supply informal care. We also assess the impact of the socioeconomic status of potential care recipients in terms of educational level and income.

## Data and method

### Data

We use linked register data for the entire non-institutionalized Swedish population in 2016, aged 65 and older (N 1,900,546). Information on home care draws from the “Register of municipal care for elderly and individuals with disabilities in accordance with the Social Services Act, (SoL)” [Registret över insatser till äldre och personer med funktionsnedsättning]. This register is maintained by the National Board of Health and Welfare. According to the Social Services Act, municipalities must report the type and extent of care services provided to citizens every month. This data allows us to investigate potential differences in service provisions dependent on the individual's demographic, socioeconomic, and health-related characteristics.

For all analyses, we include individuals 65 years or older living at home, surviving the entire year and thus potentially being eligible for home care from January 1 until December 31, 2016. We linked the home care register to the “Register of the Total Population (RTB)” at Statistics Sweden, using the personal identification number used in all Swedish administrative registers. We retrieved data on municipalities of residence, sex, and age, coded into 5-year age groups, with everyone older than 90 as a single group of oldest-old. As for socioeconomic status, we use the “Longitudinal integrated database for health insurance and labor market studies (LISA)” at Statistics Sweden. From LISA, we use the highest achieved education recorded according to the International Standard Classification of Education (ISCED)—classification system. We distinguish between primary, secondary, undergraduate, and graduate-level education. Apart from education, we also include information on the total disposable income of the individual, coded into four income levels corresponding to the quartiles of the income distribution. Finally, we used the “Dwelling register” at Statistics Sweden to distinguish older adults living alone from those that cohabitate. Most previous research has used the individual's civil status as a proxy for living arrangements, thus overestimating the proportion of individuals living alone. In our data, among those listed as unmarried, widowed, or divorced, almost one-third are cohabitating with someone. It is important to note that almost all cohabiting older adults live with their spouse/partner rather than adult children or other relatives. Sweden has one of the lowest rates of older adults living with their children in the developed world (Rodrigues et al. 2012; Iacovou and Skew 2011). Although many older adults may live near their children (Hjälm 2011), only about 1.5 percent share a household with at least one adult child (Albertini et al. 2018).

Using the “Multiple generation register,” we link the individuals to children alive in 2016 and determine if at least one of these children lives in the same municipality. In combination with the variable for living arrangements, this provides information on the potential availability of family members that can function as caregivers.

To get an estimation of the individuals' health status, we calculated the Charlson Comorbidity Index (CCI) based on the International Classification of Diseases Version 10 (ICD-10) diagnoses in the National inpatient register (NPR) (Charlson et al. 1987; Charlson et al. 1994, as presented by Brusselaers and Lagergren 2017). This version is an additive index of 14 clinically relevant co morbidities associated with increased mortality risk, including cancer, diabetes, dementia, and myocardial infarction.^7^ All primary and secondary diagnoses in the NPR within the prior ten years are considered to estimate CCI scores.^8^ We coded all individuals that had no record of any co morbidities as Healthy, those having one as Mild, two as Moderate, and three or more co morbidities as Severe.

### Statistical method

When analyzing the individual-level determinants of receiving home care and the mean monthly hours of care provided, we estimate multi-level logit and ordinary least square models of the log odds of receiving homecare and the mean monthly hours. To ensure accurate standard errors and control for variation in both outcomes across different municipalities, we include a municipal-level random intercept in our logit and ordinary least squares (OLS) models to adjust for any unobserved heterogeneity shared by individuals living in the same municipality.

The multi-level approach is motivated because we expect regional heterogeneity in home care supply as it is locally funded and administered. Of Sweden’s 290 municipalities, 59 did not report the number of hours provided. These municipalities are excluded from the analysis of the provided number of hours. In addition, we also excluded two additional municipalities from the OLS model as they exhibited markedly inflated residuals. Thus, the OLS analysis is based on 229 of Sweden's 290 municipalities. For our OLS analysis of mean monthly hours, we also condition on individuals receiving personal care or services and exclude those only having security alarms or “meals on wheels.” Our logit model of receiving home care includes 288 municipalities. We excluded two municipalities because one exhibited markedly inflated deviance residuals in our final model specification, and one did not report any information to the register in 2016. A complete list of the municipalities used to calculate the probability of receiving care and the mean monthly hours of care provided to the individual are found in the online appendix.

We choose to analyze the dichotomous outcome of having home care or not and the number of services received in terms of mean monthly hours separately because the two outcomes are not necessarily determined in the same way. First, only about 10% of all individuals in our data receive home care. Individuals excluded from home care by the municipality can, by definition, only have zero hours of care. Thus, they should not be included in the analysis of hours provided for conceptual reasons. Second, including the large share of individuals who do not receive home care creates a heavily zero-inflated dataset and a skewed distribution of the outcome variable. This would make accurate estimations of the determinants of hours provided more challenging from a statistical point of view. It would necessitate a more complex modeling approach such as zero-inflated Poisson regression rather than a more parsimonious and easily interpretable ordinary least squares regression. Robustness checks using zero-inflated models, including individuals with zero hours, do not lead to any substantive differences in conclusions (results available upon request). Therefore, we chose the more parsimonious approach and modeled the mean monthly hours using OLS only for individuals who receive some care.

We use robust standard errors to relax the assumption on the normality of residuals for the OLS model to ensure accurate estimates as the outcome is positively skewed. Our models are robust in terms of passing all standard diagnostic tests and exhibit no signs of misspecification. The final model specification was determined by likelihood ratio tests of the variable's contribution to model fit and comparison of Akaike information criterion (AIC) for alternative models (Burnham and Anderson 2004). We are using the municipality as our clustering variable allows for the intercept to vary across municipalities. In this way, we can control for unobserved heterogeneity shared by individuals living in the same municipality (Rabe-Hesketh and Skrondal, 2012a, b).

For the logit model, we report exponentiated coefficients as odds ratios exp(B), and in the OLS model, we report standard coefficients in terms of the change in mean monthly hours of a one-unit change in the independent variable. Differences in the predicted probability to have home care between individuals with different combinations of covariate values are consistently reported as an average marginal effect (AMEs) in graphs derived from the model estimates reported in Table 2. Figures use two sets of probability ranges. One for large effects ranging from 0 to 60% and one for more minor effects ranging from 0 to 15% as differences between levels would be difficult to discern whether the same range is applied for all graphs. For options available to calculate predicted probabilities from nonlinear models such as logistic regression, see, e.g., Cameron and Trivedi (2009). All estimations are calculated in Stata version 16.1.

## Results

Descriptives of how home care is distributed across the variables included in our analysis are presented in Table 1.

**Table 1:** Descriptive statistics showing relative column frequencies for the total column and relative row frequencies for the share having/not having home care *Source* Longitudinal integrated database for health insurance and labor market studies (LISA), Statistics Sweden (SCB), Inpatient-register and SoL-register, Government Board of Health and Welfare

	Total		No home care		Has home care	
	(*N* = 1 840 107)		(*N* = 1 658 605)		(*N* = 181 502)	
	N	%	N	%	N	%
*Age-group*						
65–69	551 089	29.9	540 143	98.0	10 946	2.0
70–74	521 600	28.3	502 352	96.3	19 248	3.7
75–79	338 245	18.4	311 323	92.0	26 922	8.0
80–84	225 883	12.3	186 560	82.6	39 323	17.4
85–89	137 208	7.5	89 886	65.5	47 322	34.5
90 +	66 082	3.6	28 341	42.9	37 741	57.1
*Sex*						
Men	858 499	46.7	795 841	92.7	62 658	7.3
Women	981 608	53.3	862 764	87.9	118 844	12.1
*Household type*						
Cohabiting	1 188 779	64.6	1 134 175	95.4	54 604	4.6
Living alone	651 328	35.4	524 430	80.5	126 898	19.5
*Level of education*						
Primary	625 305	34.0	537 230	85.9	88 075	14.1
Secondary	746 632	40.6	682 906	91.5	63 726	8.5
Under-graduate	191 121	10.4	178 703	93.5	12 418	6.5
Graduate level	277 049	15.1	259 766	93.8	17 283	6.2
*Income quartile*						
−25%	495 922	27.0	440 459	88.8	55 463	11.2
26–50%	513 339	27.9	431 622	84.1	81 717	15.9
51–75%	465 183	25.3	432 720	93.0	32 463	7.0
76%-	365 663	19.9	353 804	96.8	11 859	3.2
*Charlson index*						
Healthy	1 294 759	70.4	1 219 448	94.2	75 311	5.8
Mild	342 140	18.6	292 573	85.5	49 567	14.5
Moderate	131 377	7.1	100 585	76.6	30 792	23.4
Severe	71 831	3.9	45 999	64.0	25 832	36.0
*Family availability*						
No living children	330 944	18.0	296 582	89.6	34 362	10.4
Child not in same municipality	501 205	27.2	454 863	90.8	46 342	9.2
Child in same municipality	1 007 958	54.8	907 160	90.0	100 798	10.0

The share receiving home care increases sharply with age, with only 2 percent of 65–69-year old's having home care compared to 57 percent among 90 + . Almost twice as many women receive home care compared to men, and the share of having home care is about three times as large among those that live alone compared to those that cohabitate. Co morbidities are strongly associated with having home care where approximately 6 percent of those with no record of co morbidities receive home care, while this share is 36 percent among those with a severe level on our CCI measure. The share of individuals receiving home care decreases markedly as income and education increase, while there is only a weak bivariate correlation between family availability and the probability of receiving home care.

**Fig. 1:**
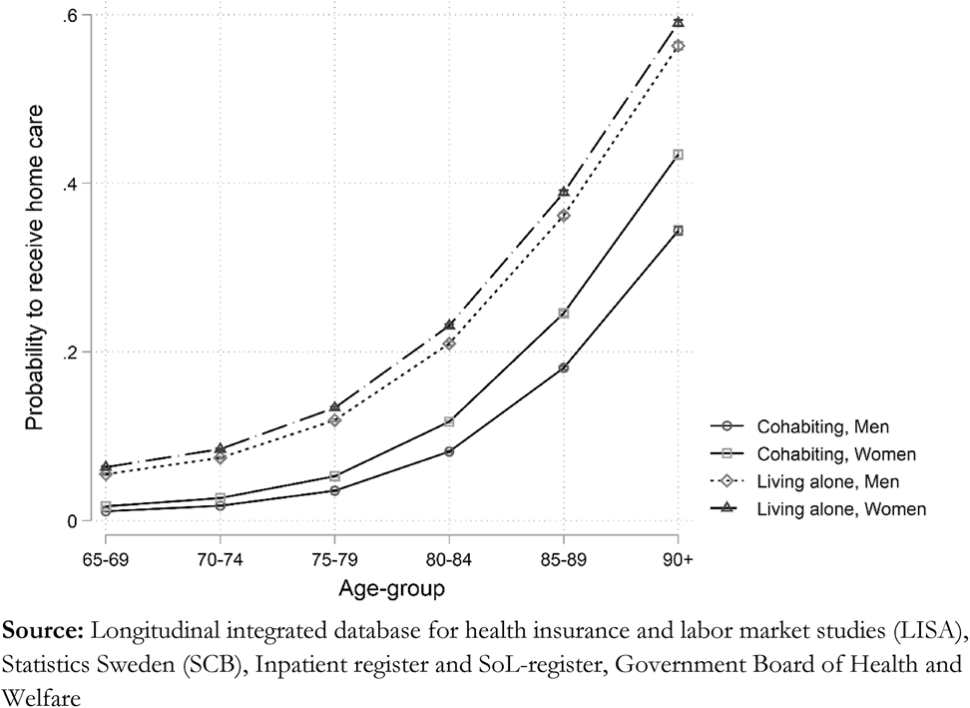
Average marginal effect of living alone by age-group, predicted probabilities

For the most part, the associations apparent in the descriptives are confirmed by our regression analysis presented in Table 2, showing the logistic regression for the probability of receiving home care. Age stands out as the most vital determinant for which the odds of having home care are approximately 11 times higher for individuals aged 80–84 and skyrocket to 73 times higher for those over 90 than those aged 65–69. Apart from age, the household type of the individual also has a strong influence. Those living alone have almost six times higher odds of receiving home care compared to cohabiting individuals. However, living arrangements depend on age and sex, where the importance of living alone decreases as individuals age. The impact of living arrangements is also different for men and women. Men are less likely than women to receive home care if they are cohabiting. This pattern is more easily observed when examining the influence of age, sex, and living arrangements on the probability scale, as an average marginal effect rather than as odds ratios (Fig. 1).

**Table 2:** Radom intercept logistic regression, odds ratios and 95%-confidence intervals [CI] to receive home care and random intercept ordinary least square regression and 95%-CI of the difference in mean number of home care hours for the Swedish population aged 65 + in 2016 *Source* Longitudinal integrated database for health insurance and labor market studies (LISA), Statistics Sweden (SCB), Inpatient-register and SoL-register, Government Board of Health and Welfare

Variables	Logit home care	OLS home care hours
	OR (95%-CI)	Beta (95%-CI)
*Age-group*		
65–69	1	0
70–74	1.70 (1.60–1.80)	− 0.38 (− 2.02–1.26)
75–79	4.00 (3.78–4.23)	− 1.29 (− 2.73–0.15)
80–84	11.04 (10.4–11.66)	− 1.49 (− 3.01–0.03)
85–89	29.51 (27.91–31.20)	− 1.32 (− 3.18–0.54)
90 +	72.81 (68.27–77.67)	4.55 (2.39–6.71)
*Charlson index*		
Healthy	1	0
Mild	3.23 (3.07–3.40)	4.59 (3.98–5.20)
Moderate	6.91 (6.50–7.34)	8.48 (7.64–9.32)
Severe	15.01 (14.02 16.08)	13.34 (12.03 14.64)
*Sex*		
Man	1	0
Women	1.42 (1.39–1.45)	2.40 (1.83–2.97)
*Household type*		
Cohabitating	1	0
Living alone	5.72 (5.47–5.99)	2.98 (1.85–4.10)
*Family availability*		
No living children	1	0
Child not in same municipality	0.80 (0.78–0.81)	− 2.83 (− 3.67 to 1.98)
Child in same municipality	0.73 (0.72–0.74)	− 1.69 (− 2.48 -0.89)
*Level of education*		
Primary	1	0
Secondary	0.92 (0.91–0.93)	− 0.68 (− 1.29 to 0.07)
Under-graduate	0.89 (0.87–0.91)	− 1.18 (− 2.19 to 0.17)
Graduate level	0.92 (0.90–0.94)	0.04 (− 0.92–1.01)
*Income quartile*		
-25%	1	0
26–50%	0.97 (0.93–1.02)	− 0.50 (− 1.24 to 0.23)
51–75%	0.45 (0.42–0.48)	0.10 (− 0.82 to 1.02)
76%-	0.28 (0.26–0.30)	2.41 (0.67–4.14)
Household type * Age-group		
Living alone * 70–74	0.85 (0.81–0.90)	
Living alone * 75–79	0.69 (0.66–0.73)	
Living alone * 80–84	0.55 (0.52–0.58)	
Living alone * 85–89	0.47 (0.45–0.49)	
Living alone * 90 +	0.45 (0.42–0.47)	
Household type * Sex		
Living alone * Women	0.76 (0.74–0.78)	
Age-group * Income quartile		
70–74 * 26–50%	1.01 (0.95–1.08)	
70–74 * 51–75%	1.29 (1.20–1.39)	
70–74 * 76%-	1.57 (1.43–1.71)	
75–79 * 26–50%	0.96 (0.90–1.02)	
75–79 * 51–75%	1.44 (1.35–1.55)	
75–79 * 76%-	2.10 (1.93–2.29)	
80–84 * 26–50%	0.95 (0.89–1.00)	
80–84 * 51–75%	1.69 (1.58–1.80)	
80–84 * 76%-	2.53 (2.32–2.75)	
85–89 * 26–50%	0.94 (0.89–1.00)	
85–89 * 51–75%	1.79 (1.67–1.92)	
85–89 * 76%-	2.91 (2.67–3.17)	
90 + * 26–50%	0.99 (0.93–1.05)	
90 + * 51–75%	1.91 (1.77–2.06)	
90 + * 76%-	2.99 (2.71–3.30)	
Charlson index * Age-group		
Mild * 70–74	0.83 (0.78–0.88)	
Mild * 75–79	0.73 (0.69–0.77)	
Mild * 80–84	0.60 (0.57–0.63)	
Mild * 85–89	0.54 (0.51–0.57)	
Mild * 90 +	0.50 (0.47–0.53)	
Moderate * 70–74	0.80 (0.74–0.86)	
Moderate * 75–79	0.60 (0.56–0.65)	
Moderate * 80–84	0.44 (0.41–0.47)	
Moderate * 85–89	0.37 (0.35–0.40)	
Moderate * 90 +	0.31 (0.29–0.34)	
Severe * 70–74	0.85 (0.78–0.92)	
Severe * 75–79	0.59 (0.54–0.63)	
Severe * 80–84	0.38 (0.36–0.41)	
Severe * 85–89	0.27 (0.25–0.29)	
Severe * 90 +	0.22 (0.20–0.24)	
Charlson index * Sex		
Mild * Women	1.12 (1.09–1.15)	
Moderate * Women	1.18 (1.14–1.22)	
Severe * Women	1.14 (1.10–1.19)	
cons	.008 (.007–.009)	23.38 (22.99 25.77)
Intra class correlation	0.02	0.07
SD (_cons)	1.32	13.34
*N*	1 840 107	132 442

**Fig. 2:**
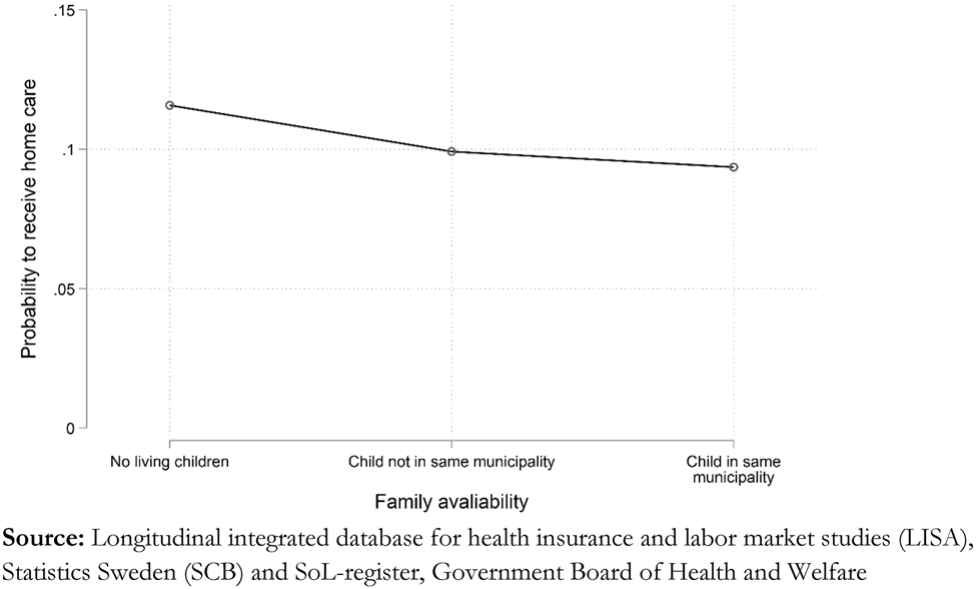
Average marginal effect of family availability, predicted probabilities.

Compared to being childless, the odds of receiving home care are reduced by 20 percent among those with children and 27 percent if they have at least one child living in the same municipality. However, when we look at the outcome as an average marginal effect shown in Fig. 2, we see that the influence of this variable is relatively modest when assessed on the absolute probability scale rather than as relative odds. Those with no children have an 11.5 percent probability of receiving care, and those who have children living in the same municipality 9 percent.

### Socioeconomic differentials

Both education and income show a clear negative association with having home care. Those having more than primary education have about 10 percent lower odds of receiving home care than those who do not. Income has a more substantial negative impact among the youngest old aged 65–69, where the top income quartile has 70 percent lower odds of having home care than those in the bottom income quartile. However, this negative effect is strongly attenuated as individuals age, as seen by the interaction between age and income. The negative interaction means that at age 80, all income groups have similar probabilities of receiving home care.

**Fig. 3:**
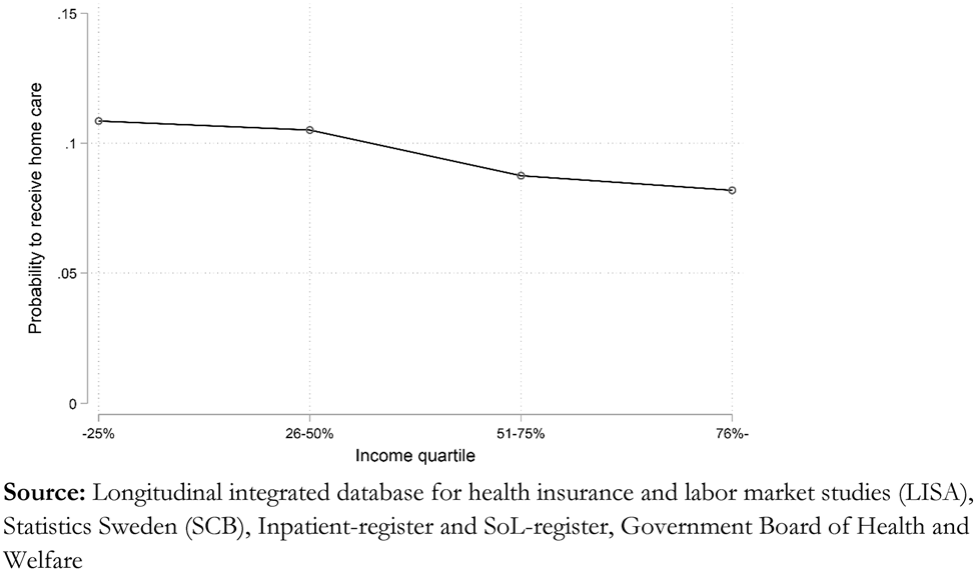
Average marginal effect of income level, predicted probabilities.

Figure 3 shows the predicted probability of receiving home care rather than the relative odds. We find that, on average, 11 percent among those in the lowest income quartile, as opposed to 8 percent among those in the highest quartile, are receiving home care when we adjust for the other covariates in the model. Thus, the average marginal effect across all age groups is not more than three percentage points.

### Health differentials

**Fig. 4:**
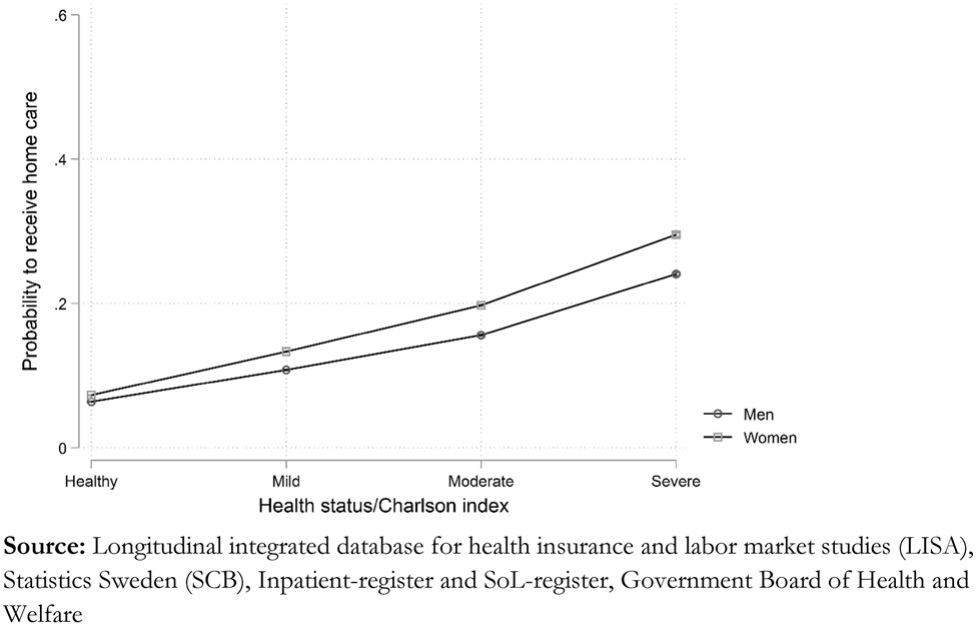
Average marginal effect of Charlson index by sex, predicted probabilities.

As measured by the CCI, poor health is, second to age, the most critical determinant for receiving home care. Individuals with severe co morbidities have almost 15 times higher odds of receiving home care than those having no record of co morbidities. However, we find that both the age and the sex of the individual modify this association. The effect of having poor health on the odds of receiving home care is stronger for women than for men. As shown in Fig. 4, the predicted probability of receiving home care among individuals with severe health problems is almost 30 percent for women and about 24 percent for men but only 6–7 percent among men and women with no record of any co morbidities.

Age also modulates the effect of having poor health, which is strongest among those aged 65–69 but declines sharply as a function of age. The odds of receiving home care for individuals with severe levels of CCI aged 90 + are only about three times as large as among those with no record of co morbidities, while those in the age group 65–69 with severe co morbidities have approximately 15 times higher odds of getting home care.

### Amount of home care

The second stage of our analysis focuses on the differences in the individual's amount of care rather than the simple distinction of who receives care or not. Table 2 shows the OLS estimates of mean monthly hours of service among home care receivers. As for the probability of receiving home care, age and co morbidity are the two strongest predictors for the number of hours received. Those aged 90 + receive 4,5 additional hours per month than the reference group aged 65–69 years. However, the increase is not linear and only becomes significant for individuals aged 90 and over.

In contrast to the probability of receiving care or not, we do not find any significant effect modification by age and sex for the amount of home care. However, in the same way, as for the probability of receiving care, severe multi-morbidity increases the support given by approximately 13 h per month. Women receive on average 2.4 h more care than men, and those living alone receive about three additional hours of care compared to those that cohabitate. Having children reduces the number of home care hours compared to those being childless. There is no evidence for a more substantial negative impact of having children in the same municipality among those with children.

Education plays a minor curvilinear role in the amount of home care received. On the other hand, income plays a statistically significant role and the association between income level and the number of home care hours follows a different gradient than the probability of receiving care. Those with the highest incomes have on average 2,4 h more care per month than those in the lowest income quartile. The positive association between income and the amount of care is the only apparent difference in the direction of effects between the OLS analysis of mean monthly hours of home care and the logistic estimates of the probability to receive care as a dichotomous outcome.

Finally, it is worth noting that despite very diverse regional pre-conditions, the municipality in which the individual lives appears to have a small or even negligible impact on the probability of receiving home care, as shown by the estimate of intraclass correlation for our random intercept models in Table 2. The unobserved heterogeneity across municipalities explains only about two percent of the total variation in the probability of receiving support. We find a slightly more significant regional variation in the mean number of hours of care, where dissimilarities across municipalities explain approximately seven percent of the total variation. So, even though it is a matter of interpretation if these regional differences are small or large, the probability of getting home care and the amount of care an individual receives are strongly determined by individual-level characteristics such as age, health, and living arrangements rather than by the municipality in which they live.

## Discussion and conclusions

The main objective of our study was to determine how home care services in contemporary Sweden are distributed with regard to individual-level factors such as health status, living arrangements, availability of family, and socioeconomic position.

Our results show that the age and health of the individual play a crucial role in both the chance of getting home care and the amount of home care received. This is well in line with previous research on smaller local data sets showing that home care is increasingly given to the oldest old, while the younger-old might have fewer needs or rely more on informal care (Johansson et al. 2018; Sundström et al. 2006; Larsson 2006; Larsson et al. 2014).

We find that the odds of getting home care among older adults with severe co morbidities are almost 15 times larger than among those with no record of co morbidities. They have on average 13 h more monthly care than healthy individuals. However, we also find that differences in the probability of receiving home care between those with and without severe co morbidities decrease with age. Including co morbidity allows us to control for differences in health caused by severe illnesses across age and socioeconomic status. However, it is essential to note that co morbidity only works as a proxy for health differentials that arguably are associated with the individual's care needs. A drawback of using this kind of administrative data is that we lack more fine-grained information of care needs, such as limitations in activities of daily living (ADL/IADL). The reduced impact of co morbidity with age probably depends on the construction of the CCI. It is based solely on hospital admissions and misses general increases in loss of function with old age. Needing more assistance with personal activities of daily living, such as clothing and feeding, does not necessarily go hand in hand with hospitalization.

The living arrangement is another essential determinant for receiving home care. Living alone increases the odds of receiving home care almost six times compared to those cohabitating. However, there is a clear difference between men and women. When living with somebody, women have a 42 percent higher odds of getting home care than cohabitating men. The combination of living arrangements and sex on the probability of receiving home care probably reflects different gender roles. When cohabitating, women may be more likely to take the role of caregivers when their partners require support than men. Additionally, men are more likely to be unable to assume a caregiver role due to impairments, as men in cohabitation tend to be older than their spouses. (Joe et al. 2019; Sigurdardottir and Kåreholt 2014; Noël-Miller 2010; Dahlberg et al. 2007; Larsson 2006; Larsson and Thorslund 2002). However, it is worth noting that we find no interaction between sex and living arrangement for the number of care hours provided. Living alone is associated with an equal increase in home care hours among both men and women, even if women, in general, have more hours than men. This underlines that the factors influencing whether care is received and how much care is allocated are necessarily not the same, showing that it is crucial to analyze these two processes separately.

The interpretation that spouses function as the most critical substitute for municipal care is reinforced by the modest impact of having children on the probability of receiving care and the amount of care. We find that having children relatively close by reduces the probability of receiving home care, although the effect is small compared to other determinants. Additionally, the effect of having children living nearby on hours provided is weak and unclear. If family support is mainly in the form of IADL, proximity between children and parents will play a less significant role. Several components in IADL can be supplied from a distance, such as contacts with authorities, ordering medicine, and taking care of economic transactions.

However, we only considered an imprecise proxy for the distance between parents and children, indicating whether they live in the same municipality. Distances between family members can be larger within a single municipality than between two other municipalities, especially if a family lives closer to a municipal border. Unfortunately, we do not have access to more precise geographic information due to the regulation of protecting the personal integrity of the research subjects. An additional limitation with the data is that we can only link the study population to close family members, i.e., partners and children, but not a wider circle of relatives. Nevertheless, given the very modest impact of having children, we conclude that any re-familization of older adults' care is primarily carried by those living in the same household. Worth noting is, however, that previous research has shown that the educational level of the child matter for the health and mortality of the parent (Meyer et al. 2019; Torssander 2013), and highly educated children more often live further away from their parents (Torssander 2014). This could potentially explain why we did not find a larger effect of proximity between parents and children.

Finally, our study shows complex relationships between education as well as personal income and home care. Socioeconomic resources are negatively associated with having home care, with income being far more critical than education. However, the negative effect is primarily a factor among the youngest old in their sixties and early seventies, while income has little to no impact among those aged over 80. A possible explanation for this age gradient is that some older adults continue to work after the average retirement age of 65. Thus, besides being healthier, they will achieve higher incomes than those who rely only on a regular pension. Given this, having a higher income is selective on better health and thus results in less need for home care.

In general, higher income levels have a negative effect on the probability of receiving home care. However, once receiving home care, income has a positive effect on the hours received each month. It is possible that, once municipal home care becomes a necessity, a high income gives the ability to pay for additional forms of support, such as cleaning services and shopping. Consequently, higher incomes reduce economic constraints to pay for higher amounts of services. Education is more likely associated with health in general. Besides having a direct effect on older adults' health, children's educational level also plays a role in the health and mortality of their parents (Meyer 2019; Leopold 2018). This effect is most substantial if children have a higher education than their parents. However, highly educated children more often live further away from their parents, so proximity and educational level may act in opposite directions (Bordone 2009).

A final issue worth commenting on is regional differences in home care. The home care model in Sweden, where the municipalities are responsible for finance, organization, and carrying out services, is heavily debated from a budget perspective. Almost all municipalities report high costs and are signaling budget cuts in their care of older adults (Plesner 2020). However, despite substantial variation in tax levels and dependency ratios between the municipalities, our estimates for regional variation in care provision and mean monthly hours are modest. Only 2% of the total variation in the probability of receiving care is explained by the municipal level clustering variable. One possible explanation for these modest regional variations could be the “Municipal financial equalization system.” This system can even out differences in budget abilities by transferring money from richer to poorer municipalities. The end goal of this system is to provide all municipalities with equal abilities to provide services, mainly regarding the care of children and older adults. However, the system has recently received sharp criticism from The Swedish National Audit Office for being insufficient. According to the audit, the system still does not fully compensate due to significant differences between municipalities' geography, inhabitants, population changes, and socioeconomic factors (Swedish National Audit Office 2019).

Regarding the strengths and limitations of the present study and potential avenues for further research, we argue that utilizing data from several different registers with national coverage is the main strength of the analysis we have presented. This allows us to produce results regarding demographic, socioeconomic, and health-related determinants of home care that can be generalized to the entire older population of Sweden rather than to smaller local samples from certain municipalities. At the same time, the use of register data also has some limitations. That we need to rely on proxies for access to family caregivers outside the household and for the individual's health status rather than ADLs/IADLs has already been mentioned. More generally, we only analyze data for 2016 and cannot say anything about potential changes over time, and naturally, our results cannot be generalized outside of the specific institutional context that the Swedish home care system represents. It is also important to note that approximately 20% of the municipalities did not report the number of home care hours provided.

Consequently, we cannot exclude the possibility of some selection bias in the data regarding the amount of care provided. Therefore, the modest heterogeneity in hours supplied should be interpreted with some caution. Given that data improve in the years to come, how equitable care is distributed between Sweden’s 290 municipalities is an important avenue for further research.

To conclude, our study shows that home care allocation in contemporary Sweden is firmly allocated to the oldest old and those with high co morbidity levels, despite contextual differences between municipalities. The observed differences between men and women, with women being more likely to receive home care, partly reflect the predominant gender roles where women function as caregivers for male partners.

Finally, the finding that single living plays a significant role in receiving home care and the extent of services implies some attention for the future. In the coming decades, the Swedish population will experience an increasing share of older adults. Recent declines in fertility (Jalovaara et al. 2018) and internationally high levels of single living among the middle-aged and the old (Padyab et al. 2019) indicate that demographic trends will not offset the increased demand for municipal services caused by an increased share of older adults. Instead, higher levels of childlessness and the internationally high share of one-person households will likely further contribute to a growing demand for home care services in Sweden.

## Supplementary Information

Below is the link to the electronic supplementary material.

Supplementary file1 (DOCX 50 KB)

## Data Availability

Data is available for research from Statistics Sweden and the National Board of Health and Welfare, Sweden, given that researchers receive ethical permission.

## Footnotes

1 The term ‘ageing in place’ implies that older people will remain in the community, either in their family homes, in homes to which they have moved in mid or later life, or in supported accommodation of some type, rather than moving into residential care. (Davey et al. 2006, p. 20).

2 For aging adults with needs of care and support, two main types exist in Sweden. *Home care*, which means that the person gets care in their own residence. It could sometimes, under special circumstances, include more advanced health care by visiting medical personnel. The other main form is in *Residential homes* (Särskilt boende***)*** for people with extensive health and social care requirements, i.e. they have major medical and social needs and require around-the-clock care.

3 The Survey of Health, Ageing and Retirement in Europe (SHARE) is a research infrastructure for studying the effects of health, social, economic and environmental policies over the life course of citizens from 28 European countries and Israel. http://www.share-project.org.

4 Activities of Daily Living (ADL) are activities in which people engage on a day-to-day basis, such as washing, toileting, dressing, feeding, mobility, and transferring. Instrumental activities of daily living (IADL) are those activities that allow an individual to live independently, such as shopping, preparing meals, housekeeping, using transportation, taking medication and managing finances and contacts with authorities.

5 The monthly fee differs slightly between municipalities, with different models applied depending on how much home care that is needed. Usually, services such as cleaning and shopping. comes as separate extra costs.

6 We use the term gender to describe the characteristics of women and men that are socially constructed, while sex refers to the biological definition of men and women which is the variable we have access to in the registers.

7 Complete list: Myocardial infarction, congestive heart failure, peripheral vascular disease, cerebrovascular disease, dementia chronic pulmonary disease, rheumatic disease, liver disease, diabetes mellitus, hemiplegia, paraplegia, renal disease, cancer, metastatic tumors, and AIDS/HIV.

8 That is, CCI for 2016 is based on hospitalizations in 2006–2015.

## Code availability

Code to reproduce analysis is available from the authors upon request.

## References

[CR1] Albertini M, Kohli M (2013). The generational contract in the family: an analysis of transfer regimes in Europe. Eur Sociol Rev.

[CR2] Albertini M, Gähler M, Härkönen J (2018). Moving back to "mamma"? Divorce, intergenerational coresidence, and latent family solidarity in Sweden. Popul Space Place.

[CR3] Arpino B, Bordone V, Balbo N (2018). Grandparenting, education and subjective well-being of older Europeans. Eur J Ageing.

[CR4] Batljan I, Lagergren M, Thorslund M (2009). Population ageing in Sweden: the effect of change in educational composition on the future number of older people suffering severe ill-health. Eur J Ageing.

[CR5] Bordone V (2009). Contact and proximity of older people to their adult children: a comparison between Italy and Sweden. Popul Space Place.

[CR6] Brusselaers N, Lagergren J (2017). The Charlson comorbidity index in registry-based research: which version to use?. Methods Inf Med.

[CR7] Burnham KP, Anderson DR (2004). Multimodel inference understanding AIC and BIC in model selection. Soc Methods Res.

[CR8] Cameron AC, Trivedi PK (2009) Microeconometrics using Stata. Stata Press

[CR9] Charlson M (1987). A new method of classifying prognostic co-morbidity in longitudinal studies: development and validation. J Cronic Diseaces.

[CR10] Charlson M, Szatrowski TP, Peterson J, Gold J (1994). Validation of a combined co-morbidity index. J Clin Epidemiol.

[CR11] Dahlberg L, Demack S, Bambra C (2007). Age and gender of informal carers: a population-based study in the UK: Age and gender of UK carers. Health Soc Care Community.

[CR12] Davey A, Johansson L, Malmberg B, Sundström G (2006). Unequal but equitable: an analysis of variations in old-age care in Sweden. Eur J Ageing.

[CR13] Fihel A, Kalbarczyk M, Nicińska A (2021). Childlessness, geographical proximity and non-family support in 12 European countries. Ageing Soc.

[CR14] Hank K, Buber I (2009). Grandparents caring for their grandchildren: findings from the 2004 survey of health, ageing, and retirement in Europe. J Fam Issues.

[CR15] Hedman NO, Johansson R, Rosenqvist U (2007). Clustering and inertia: structural integration of home care in Swedish elderly care. Int J Integr Care.

[CR16] Hjälm A (2011) A family landscape: on the geographical distances between elderly parents and adult children in Sweden. http://urn.kb.se/resolve?urn=urn:nbn:se:umu:diva-38876

[CR17] Iacovou M, Skew AJ (2011). Household composition across the new Europe: Where do the new Member States fit in?. Demogr Res.

[CR18] Jalovaara M, Neyer G, Andersson G, Dahlberg J, Dommermuth L, Fallesen P, Lappegård T (2018). Education, gender, and cohort fertility in the Nordic countries. Eur J Popul.

[CR19] Joe A, Dickins M, Enticott J, Ogrin R, Lowthian J (2019). Community-dwelling older women: the association between living alone and use of a home nursing service. J Am Med Dir Assoc.

[CR20] Johansson L, Sundström G, Malmberg B (2018). Ett halvt århundrade svensk äldreomsorg; var står stat och familj?. Tidsskrift for omsorgsforskning.

[CR21] Larsson K (2006). Care needs and home-help services for older people in Sweden: Does improved functioning account for the reduction in public care?. Ageing Soc.

[CR22] Larsson K, Thorslund M (2002). Does gender matter?: Differences in patterns of informal support and formal services in a Swedish urban elderly population. Res Aging.

[CR23] Larsson K, Thorslund M, Kåreholt I (2006). Are public care and services for older people targeted according to need? Applying the behavioural model on longitudinal data of a Swedish urban older population. Eur J Ageing.

[CR24] Larsson K, Kåreholt I, Thorslund M (2014). Care utilisation in the last years of life in Sweden: The effects of gender and marital status differ by type of care. Eur J Ageing.

[CR25] Leopold L (2018). Education and physical health trajectories in later life: a comparative study. Demography.

[CR26] Meyer AC, Brooke HL, Modig K (2019). The role of children and their socioeconomic resources for the risk of hospitalisation and mortality – a nationwide register-based study of the total Swedish population over the age 70. BMC Geriatrics.

[CR27] Neuberger FS, Preisner K (2018). Parenthood and quality of life in old age: the role of individual resources, the welfare state and the economy. Soc Ind Res.

[CR28] Noël-Miller C (2010). Longitudinal changes in disabled husbands' and wives' receipt of care. Gerontologist.

[CR29] Swedish National Audit Office. (2019). The municipal financial equalisation system – a need for more equalisation and better management (RIR 2019:29)

[CR30] Padyab M, Reher D, Requena M, Sandström G (2019). Going it alone in later life: a comparative analysis of elderly women in Sweden and Spain. J Fam Issues.

[CR31] Parker MG, Ahacic K, Thorslund M (2005). Health Changes among Swedish oldest old: prevalence rates from 1992 and 2002 show increasing health problems. J Gerontol A Biol Sci Med Sci.

[CR32] Plesner Å (2020) *Budget ur balans – en granskning av äldreomsorgens ekonomi och arbetsmiljö* [Rapport, Arena Idé, mars 2020]

[CR33] Rabe-Hesketh S, Skrondal A (2012b) Multi-level and longitudinal modeling using Stata Vol. 2 Categorical responses, counts and survival. Stata Press Publication

[CR34] Rabe-Hesketh S, Skrondal A (2012a) Multi-level and longitudinal modeling using Stata Vol. 1 Continuous responses. Stata Press Publication

[CR35] Rodrigues R, Huber M, Lamura G (Eds) (2012) Facts and figures on healthy ageing and long-term care: Europe and North America

[CR36] Sabater A, Graham E, Marshall A (2020). Does having highly educated adult children reduce mortality risks for parents with low educational attainment in Europe?. Ageing Soc.

[CR37] Savla J, Davey A, Sundström G, Zarit SH, Malmberg B (2008). Home help services in Sweden: responsiveness to changing demographics and needs. Eur J Ageing.

[CR38] Sigurdardottir SH, Kåreholt I (2014). Informal and formal care of older people in Iceland. Scand J Caring Sci.

[CR39] Socialtjänstlag (2001:453) https://rkrattsbaser.gov.se/sfst?bet=2001:453

[CR40] Statistics Sweden (2021). Demografisk försörjningskvot (R2) efter region och år. https://www.statistikdatabasen.scb.se

[CR41] Sundström G, Malmberg B, Johansson L (2006). Balancing family and state care: Neither, either or both? The case of Sweden. Ageing Soc.

[CR42] Sundström G, Herlofson K, Daatland SO, Hansen EB, Johansson L, Malmberg B, González MDP, Tortosa MÁ (2011). Diversification of old-age care services for older people: Trade-offs between coverage, diversification and targeting in European countries. J Care Serv Manag.

[CR43] Szebehely M, Dahl HM, Eriksen TR (2005). Care as employment and welfare provision: child care and elder care in Sweden at the dawn of the 21st century. Dilemmas of care in the Nordic welfare state : continuity and change.

[CR44] Torssander J (2013). From child to parent? The significance of children’s education for their parents’ longevity. Demography.

[CR45] Torssander J (2014). Adult children's socioeconomic positions and their parents' mortality: a comparison of education, occupational class, and income. Soc Sci Med.

[CR46] Ulmanen P, Szebehely M (2015). From the state to the family or to the market? Consequences of reduced residential eldercare in Sweden: from the state to the family. Int J Soc Welf.

