# Supplementary material for: Determinants of home care utilization among the Swedish old: nationwide register-based study
Source: Eur J Ageing. 2021 Dec 17;19(3):651–62. doi: 10.1007/s10433-021-00669-9 (PMC9424454; doi:10.1007/s10433-021-00669-9)
Supplement: Supplementary file 1 — Supplementary file1 (DOCX 50 KB) [file 10433_2021_669_MOESM1_ESM.docx]

**Table 1:** Swedish municipalities with indicators for available data. Municipalities marked A have requisite data and are included in logistic regression of having home care and municipalities marked with B are included in OLS regression of mean monthly hours.

| Nr. | Municipality | LOGIT | OLS |
| --- | --- | --- | --- |
| 1 | **114. UpplandsVäsby** | A |  |
| 2 | **115. Vallentuna** | A | B |
| 3 | **117. Österåker** | A | B |
| 4 | **120. Värmdö** | A |  |
| 5 | **123. Järfälla** | A | B |
| 6 | **125. Ekerö** | A |  |
| 7 | **126. Huddinge** | A | B |
| 8 | **127. Botkyrka** | A | B |
| 9 | **128. Salem** | A | B |
| 10 | **136. Haninge** | A |  |
| 11 | **138. Tyresö** | A | B |
| 12 | **139. Upplands-Bro** | A | B |
| 13 | **140. Nykvarn** | A | B |
| 14 | **160. Täby** | A |  |
| 15 | **162. Danderyd** | A |  |
| 16 | **163. Sollentuna** | A |  |
| 17 | **180. Stockholm** | A | B |
| 18 | **181. Södertälje** | A |  |
| 19 | **182. Nacka** | A |  |
| 20 | **183. Sundbyberg** | A | B |
| 21 | **184. Solna** | A | B |
| 22 | **186. Lidingö** | A | B |
| 23 | **187. Vaxholm** | A | B |
| 24 | **188. Norrtälje** | A | B |
| 25 | **191. Sigtuna** | A | B |
| 26 | **192. Nynäshamn** | A | B |
| 27 | **305. Håbo** | A | B |
| 28 | **319. Älvkarleby** | A | B |
| 29 | **330. Knivsta** | A | B |
| 30 | **331. Heby** | A | B |
| 31 | **360. Tierp** | A | B |
| 32 | **380. Uppsala** | A | B |
| 33 | **381. Enköping** | A | B |
| 34 | **382. Östhammar** | A | B |
| 35 | **428. Vingåker** | A | B |
| 36 | **461. Gnesta** | A | B |
| 37 | **480. Nyköping** | A | B |
| 38 | **481. Oxelösund** | A | B |
| 39 | **482. Flen** | A | B |
| 40 | **483. Katrineholm** | A | B |
| 41 | **484. Eskilstuna** | A |  |
| 42 | **486. Strängnäs** | A | B |
| 43 | **488. Trosa** | A |  |
| 44 | **509. Ödeshög** | A | B |
| 45 | **512. Ydre** | A |  |
| 46 | **513. Kinda** | A |  |
| 47 | **560. Boxholm** | A |  |
| 48 | **561. Åtvidaberg** | A | B |
| 49 | **562. Finspång** | A |  |
| 50 | **563. Valdemarsvik** | A |  |
| 51 | **580. Linköping** | A |  |
| 52 | **581. Norrköping** | A | B |
| 53 | **582. Söderköping** | A | B |
| 54 | **583. Motala** | A |  |
| 55 | **584. Vadstena** | A | B |
| 56 | **586. Mjölby** | A | B |
| 57 | **604. Aneby** | A | B |
| 58 | **617. Gnosjö** | A | B |
| 59 | **642. Mullsjö** | A |  |
| 60 | **643. Habo** | A | B |
| 61 | **662. Gislaved** | A | B |
| 62 | **665. Vaggeryd** | A | B |
| 63 | **680. Jönköping** | A | B |
| 64 | **682. Nässjö** | A |  |
| 65 | **683. Värnamo** | A | B |
| 66 | **684. Sävsjö** | A | B |
| 67 | **685. Vetlanda** | A | B |
| 68 | **686. Eksjö** | A | B |
| 69 | **687. Tranås** | A | B |
| 70 | **760. Uppvidinge** | A | B |
| 71 | **761. Lessebo** | A | B |
| 72 | **763. Tingsryd** | A | B |
| 73 | **764. Alvesta** | A | B |
| 74 | **765. Älmhult** | A | B |
| 75 | **767. Markaryd** | A | B |
| 76 | **780. Växjö** | A | B |
| 77 | **781. Ljungby** | A | B |
| 78 | **821. Högsby** | A | B |
| 79 | **834. Torsås** | A | B |
| 80 | **840. Mörbylånga** | A |  |
| 81 | **860. Hultsfred** | A |  |
| 82 | **861. Mönsterås** | A | B |
| 83 | **862. Emmaboda** | A | B |
| 84 | **880. Kalmar** | A | B |
| 85 | **881. Nybro** | A | B |
| 86 | **882. Oskarshamn** | A | B |
| 87 | **883. Västervik** | A | B |
| 88 | **884. Vimmerby** | A | B |
| 89 | **885. Borgholm** | A | B |
| 90 | **980. Gotland** | A | B |
| 91 | **1060. Olofström** | A | B |
| 92 | **1080. Karlskrona** | A | B |
| 93 | **1081. Ronneby** | A | B |
| 94 | **1082. Karlshamn** | A | B |
| 95 | **1083. Sölvesborg** | A | B |
| 96 | **1214. Svalöv** | A |  |
| 97 | **1230. Staffanstorp** | A | B |
| 98 | **1231. Burlöv** | A | B |
| 99 | **1233. Vellinge** | A |  |
| 100 | **1256. ÖstraGöinge** | A | B |
| 101 | **1257. Örkelljunga** | A | B |
| 102 | **1260. Bjuv** | A |  |
| 103 | **1261. Kävlinge** | A |  |
| 104 | **1262. Lomma** | A |  |
| 105 | **1263. Svedala** | A | B |
| 106 | **1264. Skurup** | A | B |
| 107 | **1265. Sjöbo** | A | B |
| 108 | **1266. Hörby** | A | B |
| 109 | **1267. Höör** | A | B |
| 110 | **1270. Tomelilla** | A | B |
| 111 | **1272. Bromölla** | A | B |
| 112 | **1273. Osby** | A | B |
| 113 | **1275. Perstorp** | A | B |
| 114 | **1276. Klippan** | A | B |
| 115 | **1277. Åstorp** | A | B |
| 116 | **1278. Båstad** | A | B |
| 117 | **1280. Malmö** | A | B |
| 118 | **1281. Lund** | A | B |
| 119 | **1282. Landskrona** | A |  |
| 120 | **1283. Helsingborg** | A | B |
| 121 | **1284. Höganäs** | A | B |
| 122 | **1285. Eslöv** | A | B |
| 123 | **1286. Ystad** | A | B |
| 124 | **1287. Trelleborg** | A | B |
| 125 | **1290. Kristianstad** | A | B |
| 126 | **1291. Simrishamn** | A | B |
| 127 | **1292. Ängelholm** | A |  |
| 128 | **1293. Hässleholm** | A | B |
| 129 | **1315. Hylte** | A | B |
| 130 | **1380. Halmstad** | A | B |
| 131 | **1381. Laholm** | A |  |
| 132 | **1382. Falkenberg** | A |  |
| 133 | **1383. Varberg** | A | B |
| 134 | **1384. Kungsbacka** | A | B |
| 135 | **1401. Härryda** | A | B |
| 136 | **1402. Partille** | A | B |
| 137 | **1407. Öckerö** | A | B |
| 138 | **1415. Stenungsund** | A | B |
| 139 | **1419. Tjörn** | A | B |
| 140 | **1421. Orust** | A | B |
| 141 | **1427. Sotenäs** | A | B |
| 142 | **1430. Munkedal** | A | B |
| 143 | **1435. Tanum** | A | B |
| 144 | **1438. Dals-Ed** | A | B |
| 145 | **1439. Färgelanda** | A |  |
| 146 | **1440. Ale** | A | B |
| 147 | **1441. Lerum** | A | B |
| 148 | **1442. Vårgårda** | A | B |
| 149 | **1443. Bollebygd** | A | B |
| 150 | **1444. Grästorp** | A | B |
| 151 | **1445. Essunga** | A | B |
| 152 | **1446. Karlsborg** | A | B |
| 153 | **1447. Gullspång** | A | B |
| 154 | **1452. Tranemo** | A | B |
| 155 | **1460. Bengtsfors** | A | B |
| 156 | **1461. Mellerud** | A | B |
| 157 | **1462. LillaEdet** | A | B |
| 158 | **1463. Mark** | A | B |
| 159 | **1465. Svenljunga** | A |  |
| 160 | **1466. Herrljunga** | A | B |
| 161 | **1470. Vara** | A | B |
| 162 | **1471. Götene** | A | B |
| 163 | **1472. Tibro** | A | B |
| 164 | **1473. Töreboda** | A | B |
| 165 | **1480. Göteborg** | A | B |
| 166 | **1481. Mölndal** | A | B |
| 167 | **1482. Kungälv** | A | B |
| 168 | **1484. Lysekil** | A | B |
| 169 | **1485. Uddevalla** | A | B |
| 170 | **1486. Strömstad** | A | B |
| 171 | **1487. Vänersborg** | A |  |
| 172 | **1488. Trollhättan** | A | B |
| 173 | **1489. Alingsås** | A | B |
| 174 | **1490. Borås** | A | B |
| 175 | **1491. Ulricehamn** | A | B |
| 176 | **1492. Åmål** | A | B |
| 177 | **1493. Mariestad** | A | B |
| 178 | **1494. Lidköping** | A | B |
| 179 | **1495. Skara** | A | B |
| 180 | **1496. Skövde** | A | B |
| 181 | **1497. Hjo** | A | B |
| 182 | **1498. Tidaholm** | A | B |
| 183 | **1499. Falköping** | A | B |
| 184 | **1715. Kil** | A | B |
| 185 | **1730. Eda** | A | B |
| 186 | **1737. Torsby** | A | B |
| 187 | **1760. Storfors** | A |  |
| 188 | **1761. Hammarö** | A | B |
| 189 | **1762. Munkfors** | A | B |
| 190 | **1763. Forshaga** | A | B |
| 191 | **1764. Grums** | A | B |
| 192 | **1765. Årjäng** | A | B |
| 193 | **1766. Sunne** | A | B |
| 194 | **1780. Karlstad** | A | B |
| 195 | **1781. Kristinehamn** | A |  |
| 196 | **1782. Filipstad** | A | B |
| 197 | **1783. Hagfors** | A |  |
| 198 | **1784. Arvika** | A |  |
| 199 | **1785. Säffle** | A | B |
| 200 | **1814. Lekeberg** | A | B |
| 201 | **1860. Laxå** | A | B |
| 202 | **1861. Hallsberg** | A | B |
| 203 | **1862. Degerfors** | A | B |
| 204 | **1863. Hällefors** | A | B |
| 205 | **1864. Ljusnarsberg** | A | B |
| 206 | **1880. Örebro** | A | B |
| 207 | **1881. Kumla** | A | B |
| 208 | **1882. Askersund** | A | B |
| 209 | **1883. Karlskoga** | A | B |
| 210 | **1884. Nora** | A | B |
| 211 | **1885. Lindesberg** | A | B |
| 212 | **1904. Skinnskatteberg** | A | B |
| 213 | **1907. Surahammar** | A |  |
| 214 | **1960. Kungsör** | A | B |
| 215 | **1961. Hallstahammar** | A | B |
| 216 | **1962. Norberg** | A |  |
| 217 | **1980. Västerås** | A |  |
| 218 | **1981. Sala** | A |  |
| 219 | **1982. Fagersta** | A |  |
| 220 | **1983. Köping** | A |  |
| 221 | **1984. Arboga** | A | B |
| 222 | **2021. Vansbro** | A | B |
| 223 | **2023. Malung-Sälen** | A | B |
| 224 | **2026. Gagnef** | A | B |
| 225 | **2029. Leksand** | A | B |
| 226 | **2031. Rättvik** | A | B |
| 227 | **2034. Orsa** | A | B |
| 228 | **2039. Älvdalen** | A | B |
| 229 | **2061. Smedjebacken** | A |  |
| 230 | **2062. Mora** | A | B |
| 231 | **2080. Falun** | A | B |
| 232 | **2081. Borlänge** | A |  |
| 233 | **2082. Säter** | A | B |
| 234 | **2083. Hedemora** | A | B |
| 235 | **2084. Avesta** | A | B |
| 236 | **2085. Ludvika** | A | B |
| 237 | **2101. Ockelbo** | A | B |
| 238 | **2104. Hofors** | A | B |
| 239 | **2121. Ovanåker** | A | B |
| 240 | **2132. Nordanstig** | A | B |
| 241 | **2161. Ljusdal** | A | B |
| 242 | **2180. Gävle** | A | B |
| 243 | **2181. Sandviken** | A | B |
| 244 | **2182. Söderhamn** | A |  |
| 245 | **2183. Bollnäs** | A | B |
| 246 | **2184. Hudiksvall** | A | B |
| 247 | **2260. Ånge** | A |  |
| 248 | **2262. Timrå** | A | B |
| 249 | **2280. Härnösand** | A | B |
| 250 | **2281. Sundsvall** | A | B |
| 251 | **2282. Kramfors** | A | B |
| 252 | **2283. Sollefteå** | A |  |
| 253 | **2284. Örnsköldsvik** | A | B |
| 254 | **2303. Ragunda** | A | B |
| 255 | **2305. Bräcke** | A | B |
| 256 | **2309. Krokom** | A |  |
| 257 | **2313. Strömsund** | A | B |
| 258 | **2321. Åre** | A | B |
| 259 | **2326. Berg** | A | B |
| 260 | **2361. Härjedalen** | A | B |
| 261 | **2380. Östersund** | A |  |
| 262 | **2401. Nordmaling** | A |  |
| 263 | **2403. Bjurholm** | A |  |
| 264 | **2404. Vindeln** | A |  |
| 265 | **2409. Robertsfors** | A |  |
| 266 | **2417. Norsjö** | A | B |
| 267 | **2418. Malå** | A | B |
| 268 | **2421. Storuman** | A | B |
| 269 | **2422. Sorsele** |  |  |
| 270 | **2425. Dorotea** | A | B |
| 271 | **2460. Vännäs** | A | B |
| 272 | **2462. Vilhelmina** | A | B |
| 273 | **2463. Åsele** | A | B |
| 274 | **2480. Umeå** | A | B |
| 275 | **2481. Lycksele** | A | B |
| 276 | **2482. Skellefteå** | A | B |
| 277 | **2505. Arvidsjaur** | A | B |
| 278 | **2506. Arjeplog** | A | B |
| 279 | **2510. Jokkmokk** | A | B |
| 280 | **2513. Överkalix** | A | B |
| 281 | **2514. Kalix** | A | B |
| 282 | **2518. Övertorneå** | A | B |
| 283 | **2521. Pajala** |  |  |
| 284 | **2523. Gällivare** | A | B |
| 285 | **2560. Älvsbyn** | A | B |
| 286 | **2580. Luleå** | A | B |
| 287 | **2581. Piteå** | A | B |
| 288 | **2582. Boden** | A | B |
| 289 | **2583. Haparanda** | A | B |
| 290 | **2584. Kiruna** | A | B |
| Total |  | 288 | 229 |
